# Editorial: Genetics of inflammatory and immune diseases

**DOI:** 10.3389/fgene.2023.1355794

**Published:** 2024-01-08

**Authors:** Tianyu Wang, Yunqing Ren, Xianyong Yin, Yonghu Sun

**Affiliations:** ^1^ Hospital for Skin Diseases, Shandong First Medical University, Jinan, Shandong, China; ^2^ Shandong Provincial Institute of Dermatology and Venereology, Shandong Academy of Medical Sciences, Jinan, Shandong, China; ^3^ Department of Dermatology, Children’s Hospital, School of Medicine, Zhejiang University, Hangzhou, Zhejiang, China; ^4^ Department of Biostatistics, University of Michigan, Ann Arbor, MI, United States

**Keywords:** inflammatory and immune diseases, susceptibility, genome-wide association study, high-throughput sequencing, integrative study, mechanism

## 1 Introduction

Inflammatory and immune diseases encompass a spectrum of conditions, including inflammatory bowel disease, systemic lupus erythematosus, rheumatoid arthritis, psoriasis, and atopic dermatitis (Hodson, 2016; Sroka-Tomaszewska and Magdalena, 2021; Kiriakidou and Ching, 2020). These disorders are characterized by presumed pathomechanisms involving inflammatory and immune systems, frequently exhibiting a significant genetic predisposition. Over the past few decades, genetic studies, particularly genome-wide association studies (GWAS), have identified hundreds of genetic variants robustly associated with these diseases (Klein et al., 2005). The rapid accumulation of GWAS data has broadened opportunities for biologists to discover new disease-associated variants and gain insights into the mechanisms underlying complex human diseases (Wang et al., 2016; Sun et al., 2019; Wang et al., 2021). Although GWAS is a powerful tool, it encounters challenges in precisely pinpointing candidate disease risk genes. In response to these challenges, several post-GWAS methods have emerged, including Transcriptome-Wide Association Studies (TWAS) (Gusev et al., 2016) and Mendelian Randomization (MR) (Bowden and Holmes, 2019). These methods serve as potent tools for identifying candidate disease risk genes, providing benefits such as enhanced statistical power, improved interpretability, and reduced computational costs. Importantly, the exploration of genetic mechanisms (Stikker et al., 2023; Sun et al., 2016), experimental integration research (Xia et al., 2022), multi-omics studies (Husin et al., 2017), and advancements in sequencing technology (Mi et al., 2022) has further contributed to new biological insights into the mechanisms of inflammation and immune disorders. These findings have the potential to identify drug targets of interest.

## 2 Overview of contributions

The identification of disease-causing variants is crucial for enhancing our understanding of disease pathogenesis and expediting the discovery of diagnostic biomarkers for inflammatory and immune disorders. Fang et al. reported a case study revealing a novel heterozygous variant (c.934G>T, p.Glu312Ter) in the MSN gene associated with X-linked moesin-associated immunodeficiency (X-MAID), identified through whole exome sequencing (WES) and trio analysis. This study broadened the spectrum of MSN mutations linked to immunodeficiency. Li et al. conducted an integrated bioinformatics analysis of 30 patients with systemic lupus erythematosus (SLE) to identify key genes in CD4^+^ T cells, uncovering six novel biomarkers that could contribute to the diagnosis and treatment of SLE. Fu et al. identified immune biomarkers associated with basement membranes in idiopathic pulmonary fibrosis (IPF) through integrated bioinformatics and Pan-cancer analysis. The study pinpointed immune-related hub genes that could serve as potential targets for intervention in various diseases, including IPF and cancer. Additionally, Tang et al. utilized genome sequencing analysis (WGS), meta-analyses, and replication analyses to discover a rare gain-of-function frameshift variant in SIRPB1 associated with Crohn’s disease (CD) in Han Chinese patients. The study also investigated the functional mechanism of SIRPB1 and its downstream inflammatory pathways in CD.

The integration of experimental studies with multi-omics studies holds immense potential for unraveling the intricate mechanisms underlying diseases. Yin et al. integrated experimental and transcriptomic approaches, revealing the involvement of interleukin enhancer binding factor 2 (ILF2) and KLHDC7B-DT in the hyperproliferation of keratinocytes and skin inflammation in psoriasis. Notably, ILF2 operates in a KLHDC7B-DT-dependent manner. Similarly, Yan et al. integrated experimental and transcriptomic data to elucidate the potential involvement of serum extracellular vesicles (EVs) in the activation of keratinocytes through loaded miRNAs in psoriasis. Specific miRNAs, such as miR-1305 and miR-6785-5p within serum EVs, were identified as potential contributors to psoriasis. Duan et al. adopted an integrative approach, combining experimental and bioinformatics analyses of 38 samples, to reveal a potential regulatory mechanism of m6A modification in the immune microenvironment of osteoarthritis (OA). This not only provides insights for OA treatment but also addresses a research gap in this field. Furthermore, Ribeiro et al. employed an integrated approach combining experimental and bioinformatics analyses to reveal the global haplotype-specific 3-dimensional chromatin looping architecture. This architecture significantly influences local allelic BLK and FAM167A gene expression, offering mechanistic details on how regional variants controlling the BLK promoter may impact disease risk.

The current study explores the genetic associations and pathogenesis of inflammatory and immune diseases through the application of GWAS and other analytical approaches. Elghzaly et al. performed a GWAS in an admixed Egyptian population, revealing novel novel genetic associations and providing insights into the pathogenesis of SLE. Their work contributes to an enhanced understanding of the genetic factors underlying this autoimmune condition. In a related study, Zhang et al. employed multifactor dimensionality reduction (MDR) analysis and meta-analysis techniques to elucidate the interaction between ERAP1 and IFIH1 in influencing the development of psoriasis. This approach provides valuable insights into the complex interplay of genetic factors contributing to the pathophysiology of psoriasis. Bui et al. conducted an investigation into the contributions of both HLA and non-HLA factors to clinical phenotypes within the Newfoundland psoriasis cohort. Utilizing a partitioned 88-loci psoriasis Genetic Risk Score (GRS), they successfully clarified the intricate relationship between HLA and non-HLA components of the GRS with crucial clinical features of psoriasis.

Addressing the challenging task of distinguishing causal signals from mere associations in GWAS poses a substantial challenge. To address this challenge, various methods including fine mapping, MR, and TWAS have emerged, offering avenues to translate GWAS findings into a functional understanding of associated traits. Lee et al. employed meticulous fine mapping to identify the MHC locus variant linked to late-onset asthma across diverse race/ethnicity-stratified populations. This precision enhances our understanding of the genetic basis of asthma and holds promise for the development of precise, targeted treatments tailored to specific demographic groups. Zhao et al. employed a different approach by utilizing circulating cytokines and conducting a two-sample MR analysis to identify causal cytokines associated with the risk of psoriasis vulgaris. This unveiled potential therapeutic targets and shed light on the underlying genetic factors contributing to the development of this dermatological condition. Yang et al. investigated genetically regulated 25OHD concentrations through a meta-analysis and MR study, establishing a causal link between genetically regulated 25OHD concentrations and the risk of chronic obstructive pulmonary disease (COPD). This insight provides a foundation for understanding the genetic underpinnings of COPD and may inform preventive strategies. Zhu et al. conducted a comprehensive strategy involving TWAS and multi-tissue interaction analyses. Their work unveiled candidate susceptibility genes related to respiratory infectious diseases, offering valuable insights at the genetic level and potentially informing future interventions and therapies.

Numerous studies highlighted in this Research Topic have explored diverse phenotypic anchoring strategies to establish genotype-phenotype associations in the realm of inflammatory and immune diseases. In a comprehensive genotype-phenotype association study, Chen et al. employed a multifaceted approach, incorporating clinical characteristics, *in silico* analysis, and intervention. Their investigation led to the identification of two novel compound heterozygous mutations in TTC7A, a groundbreaking discovery marking the first instance of these mutations observed in mainland China. These mutations were associated with neonatal-onset Inflammatory Bowel Disease-Combined Immunodeficiency (IBD-CID) and linked to a poor prognosis. Similarly, Xu et al. delved into the realm of congenital pseudarthrosis of the tibia (CPT) combined with neurofibromatosis type 1, employing a novel NF1 mutation. Their study utilized a combination of clinical characteristics and *in silico* analysis to unravel the intricacies of this distinctive genotype-phenotype association. This research adds valuable insights into the understanding of the complex interplay between genetic mutations and clinical manifestations in the realm of inflammatory and immune diseases.

Mini-reviews summarizing reported genetic variants, especially in the realm of inflammatory and immune diseases, serve as a valuable approach for hypothesis generation and deepening our understanding of underlying molecular mechanisms. Wang et al. conducted a review focusing on the roles of the AIM2 gene and AIM2 inflammasome in the pathogenesis and treatment of psoriasis. This mini-review aims to provide not only a comprehensive overview but also new insights into the functions of the AIM2 gene and AIM2 inflammasome for psoriasis.

## 3 Conclusion

Collectively, these articles unveil the genetic foundations of inflammatory and immune diseases. They not only elucidate the mechanisms underlying genetic associations but also uncover new therapeutic targets through the comprehensive application of GWAS, both analytically and experimentally. Post-GWAS, these studies integrate analytics and experiments to deepen our understanding of genetic mechanisms, leveraging advanced sequencing technologies. In doing so, these contributions stand as valuable knowledge resources for future medical research and clinical applications. By offering insights into the intricate genetic landscape of inflammatory and immune diseases, these studies contribute significantly to the ongoing advancement of medical knowledge, fostering the potential for innovative treatments and improved clinical outcomes.
